# Pattern and quality of care of cancer pain management. Results from the Cancer Pain Outcome Research Study Group

**DOI:** 10.1038/sj.bjc.6605053

**Published:** 2009-04-28

**Authors:** G Apolone, O Corli, A Caraceni, E Negri, S Deandrea, M Montanari, M T Greco

**Affiliations:** 1 Center for the Evaluation and Research on Pain (CERP) Department of Oncology, Istituto di Ricerche Farmacologiche ‘Mario Negri’, Milan, Italy; 2 Palliative Care Unit, Department of Anesthesiology and Palliative Care, Fondazione IRCCS Istituto Nazionale dei Tumori, Milan, Italy; 3 Institute of Medical Statistics and Biometry, University of Milano, Milan, Italy

**Keywords:** cancer pain, opioids, quality, PMI

## Abstract

Most patients with advanced or metastatic cancer experience pain and despite several guidelines, undertreatment is well documented. A multicenter, open-label, prospective, non-randomised study was launched in Italy in 2006 to evaluate the epidemiology, patterns and quality of pain care of cancer patients. To assess the adequacy of analgesic care, we used a standardised measure, the pain management index (PMI), that compares the most potent analgesic prescribed for a patient with the reported level of the worst pain of that patient together with a selected list of clinical indicators. A total of 110 centres recruited 1801 valid cases. 61% of cases were received a WHO-level III opioid; 25.3% were classified as potentially undertreated, with wide variation (9.8–55.3%) according to the variables describing patients, centres and pattern of care. After adjustment with a multivariable logistic regression model, type of recruiting centre, receiving adjuvant therapy or not and type of patient recruited (new or already on follow-up) had a significant association with undertreatment. Non-compliance with the predefined set of clinical indicators was generally high, ranging from 41 to 76%. Despite intrinsic limitations of the PMI that may be considered as an indicator of the poor quality of cancer pain care, results suggest that the recourse to WHO third-level drugs still seems delayed in a substantial percentage of patients. This delay is probably related to several factors affecting practice in participating centres and suggests that the quality of cancer pain management in Italy deserves specific attention and interventions aimed at improving patients’ outcomes.

Pain is a major problem for patients with cancer. Meta-analyses report that most patients with advanced or metastatic cancer experience pain (Hearn and Higginson, 2003; van den Beuken-van Everdingen *et al*, 2007). Although several guidelines for cancer pain management have been published since 1986 (Jacox *et al*, 1994; WHO, 1996; Hanks *et al*, 2001; Cohen *et al*, 2003; SIGN, 2008), undertreatment is well documented and can reach 82% of patients in some settings (Di Maio *et al*, 2004). A recent systematic review based on 26 papers published from 1994 to 2007 suggests that nearly one of two patients with cancer pain is undertreated with a wide variability across studies and settings (Deandrea *et al*, 2008). Undertreatment is usually attributed to inappropriate use of opioids on account of several reasons related to the healthcare provider, patient, family, institution and society (Maltoni, 2008), where fear of opioids may be the most important determinant from the patient’s point of view (Reid *et al*, 2008).

In Italy, opioid consumption rates are yet among the lowest in Europe (De Conno *et al*, 2005), although last year there was a small increase, mainly because of a change in the type of drugs prescribed than to an increase in the number of patients treated (Osservatorio Nazionale sull’impiego dei farmaci, 2007). As recently pointed out, poor information and communication may be a further barrier to a correct access to analgesic and palliative care and to hospice admission (Harrington and Smith, 2008).

To improve the quality of cancer pain management, the Mario Negri Institute implemented a project on 2004 in Italy (Apolone *et al*, 2004, 2006a), and an Outcome Research study was launched in 2006. The objectives were (a) to describe a large cohort of cancer patients in terms of pain characteristics, patterns of care and patient-reported outcomes; (b) to assess the quality of analgesic treatments in terms of congruence between the reported level of pain intensity of patients and the potency of the prescribed analgesic drug; (c) to compare the effects of various analgesic options, using appropriate statistical methods, such as the propensity score. This study reports on details about the design of the study, the type of patients recruited, the analgesic drugs prescribed and the quality of analgesic care administered.

## Patients and methods

### Study design and patients

Background and methods are described elsewhere (Apolone *et al*, 2006a, 2006b, 2008). Briefly, the data reported here were collected as part of a multicentre, open-label, prospective, non-randomised study. Each centre admitted up to 25 patients with diagnostic evidence of advanced/metastatic solid tumour; persistent pain, of any degree of intensity related to cancer, requiring or already on analgesic treatment; age ⩾18 years; life expectancy more than 1 month; and able to read, understand and provide informed consent to participate.

After enrolment/inclusion, the following screening assessments were carried out and recorded weekly for the first month, with a final visit at week 12 (at the end of the study): (a) medical history including cancer history, (b) physical examination, (c) record of medications and recent therapies, including analgesic consumption, (d) pain and symptom assessment, (f) patients’ and physicians’ satisfaction with pain treatment and (g) patient's self-reported quality of life.

### Outcomes and endpoints

Patients’ and physicians’ reports were collected using standardised forms at scheduled visits. Self-administered questionnaires were completed when the patient attended regular visits at the centre or during admission or at home depending on the setting of care. Investigators recorded information about patients and disease, pain medications and type and number of rescue doses in a case report form.

Pain characteristics (intensity, relief and so on) were the primary outcome measures. Other patient-reported outcomes were collected too, such as satisfaction with care, quality of life and symptoms. Pain was measured using five items from the Italian version of the Brief Pain Inventory (Caraceni *et al*, 1996) assessing intensity of worst, present, least and average pain and pain relief with an 11-point numerical rating scale.

We used two methods to evaluate the analgesic undertreatment. First, we applied the pain management index (PMI), developed by Cleeland *et al* (1994). According to the World Health Organisation's (WHO) guidelines for the management of pain in cancer, treatment is considered adequate when there is congruence between the reported level of pain of the patient and the potency of the prescribed analgesic drug (its place on the WHO analgesic ladder; WHO, 1996). The PMI compares the most potent analgesic prescribed for a patient with the reported level of the worst pain of that patient.

**Figure 1:**
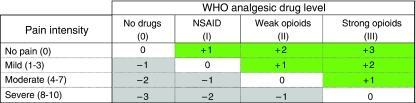
Pain management index.

To construct the index, we determined which of four levels of analgesic drug therapy was the most potent one used: 0, no analgesic drug; 1, a non-opioid (e.g., a non-steroidal anti-inflammatory drug); 2, a weak opioid (e.g., codeine or tramadol); and 3, a strong opioid (e.g., morphine, fentanyl, buprenorphine, oxycodone and so on). We then determined the patient's level of pain from the Brief Pain Inventory (1–3, mild; 4–7, moderate; 8–10, severe). No pain was scored as 0; mild pain, 1; moderate pain, 2; and severe pain, 3. The PMI, computed by subtracting the pain level from the analgesic level, ranges from −3 (a patient with severe pain receiving no analgesic drugs) to +3 (a patient receiving morphine or an equivalent and reporting no pain). Negative scores are considered to indicate pain undertreatment, and scores of 0 or higher are considered a conservative indicator of acceptable treatment (see Figure 1).

As the PMI provides only a rough estimate of how pain is treated in a sample taking into account only some attributes of the pain characteristics (Deandrea *et al*, 2008), we also assembled a list of clinical indicators to capture the appropriateness of the analgesic care delivered more directly. Operationally, we identified four specific clinical conditions where there was evidence that pain should be treated with a specific approach according to available guidelines: presence of episodes of breakthrough pain to be treated with a strong opioid as rescue/escape therapy, presence of neuropathic pain to be treated with a specific adjuvant drug, pain with intensity higher than 7 points, calling for a strong opioid as around-the-clock therapy, and presence of bone metastasis to be treated with bisphosphonates, (Hanks *et al*, 2001; Wong and Wiffen, 2002; CEVEAS, 2004; SIGN, 2008). Then, we estimated the proportion of patients in each group who did not receive the recommended therapy, as additional indicators of the quality of analgesic therapy. Frequencies were then associated with some variables, such as type of patient and recruiting centre.

Given the large number of centres, we used a web-based system to optimise the handling of all aspects of the clinical study, including data entry, quality assurance and validation. A general data entry engine for clinical trials developed by the Mario Negri Institute was used, which was compliant with current laws concerning ethical and regulatory issues (Clivio *et al*, 2006). Before implementation, it was tested with a pilot feasibility study in 130 centres in 2005.

### Statistical analysis

On the basis of the literature and the results of the pilot study in 2005, we assumed that 100 centres during a 2-month inception period (recruitment) could see and evaluate up to 2500 eligible cases. We also assumed that at least half would already be receiving a WHO level III treatment and most of the others would eventually need a WHO level III analgesic during the longitudinal evaluation period.

There were no formal estimates of the proportion of cases who might be classified as undertreated at inclusion, although, on the basis of a systematic review of the literature pertaining to the PMI (Deandrea *et al*, 2008), it was expected that up to 40% of patients might be receiving, at the time of study inclusion, an analgesic treatment not adequate to their intensity of pain. All patients enrolled in the study and eligible were included in the present analysis.

In the descriptive analysis, absolute frequency was used for categorical variables and central trend and dispersion measurements (mean, median and s.d.) for quantitative continuous variables. When comparing groups, *χ*^2^-tests for associations were used for categorical variables, such as whether or not patients experienced a given event. For continuous variables, such as mean differences in pain intensity, *t*-tests or one-way-analysis of variance, were used. For binary dependent variables, such as whether or not the patient had an analgesic undertreatment according to the PMI, estimates of association with potential independent variables were expressed in terms of odds ratios (ORs). After univariate analysis, a logistic model was fitted using maximum likelihood estimation to express the odds of each variable relative to the reference category, after adjustment for all other covariates. For example, the risk of being classified as undertreated for a patient with 70 years of age or more was compared to one younger than 51 years, given the same sex, type of cancer and analgesic adjuvant therapy. In addition, 95% confidence intervals of the ORs were computed.

Given the observational nature of the study, the relatively large sample size and the number of statistical tests, *P*-values must be considered with cautions as merely suggestive of a trend.

### Ethical considerations

The study complied with Italian requirements for observational studies. The protocol was approved by the local research ethics committees of participating centres. All patients gave written informed consent to participate in the study. The full study protocol was published in an open-access journal before the study started (Apolone *et al*, 2006b).

## Results

**Figure 2:**
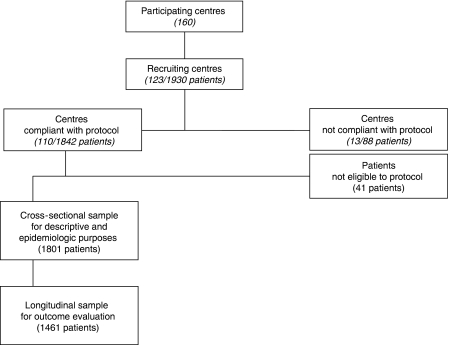
Synopsis of the stages of the study.

As shown in Figure 2, 110 centres recruited 1801 valid cases that constitute the baseline cross-sectional sample, from February 2006 to March 2007. There were 1461 patients with complete data at 28 days and they form the longitudinal sample. This study focuses on the baseline sample.

As shown in Table 1, patients recruited were more frequently male, and had severe pain (mean worst pain at baseline 6.8). Half had bone metastasis, episodes of breakthrough pain and were still on active anticancer treatment. The most frequent primary cancers were lung, breast and colorectal cancers. Nearly two-thirds were recruited by oncologic centres, and the majority were not aware of their prognosis. Most had already been admitted at the centre when enrolled in the study. In all, 61% of patients were received a WHO-level III opioid, fentanyl and buprenorphine transdermal delivery systems being the most widely prescribed (in about 39% of cases); 47% received some kind of rescue/escape therapy, which was an anti-inflammatory drug in 53% of cases; oral morphine was the most widely prescribed strong opioid as recue therapy. A total of 60% also received some kind of adjuvant analgesic therapy.

We first assembled the PMI using all four items of the Brief Pain Inventory. Table 2 shows, as expected, that different pain items generated different undertreatment estimates (8.9–25.3%). As recommended by Cleeland *et al* (1994), we used the worst pain item as main outcome. According to the literature suggesting wide variations on the basis of several variables related to patients, centres and settings (Deandrea *et al*, 2008), we first conducted a bivariate analysis to identify the potential predictors of undertreatment, then a multivariable analysis to estimate the effect of each potential confounder on the association between predictors and occurrence of undertreatment. Tables 3, 4 and 5 report the results of both analyses.

**Figure 3:**
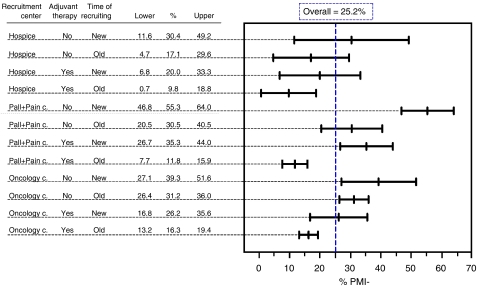
Percentages (95% confidence interval) of patients with negative PMI in all the combinations among levels of recruiting centres, adjuvant therapy and time of recruiting.

When patients were classified according to the time of the follow-up or treatment before inclusion (new incident cases *vs* patients already in follow-up at the centre), there was a clear tendency: the proportion of undertreatment ranged from 44.7 to 20.2% with a consistent and statistically significant gradient (*P*<0.0001). Among the other potential predictors tested with univariate analyses, the absence of bone metastasis and ongoing chemotherapy, being recruited in pain and palliative centres, having a colorectal cancer, and not receiving adjuvant analgesic therapy, were significantly associated with a higher probability of being classified as undertreated (OR=>1.2 and *P*<0.05). After multivariable logistic regression, only type of recruiting centre (*P*<0.01), receiving adjuvant therapy or not (*P*<0.001), and type of patient recruited (new or already on follow-up; *P*<0.01) showed a significant association with undertreatment. To describe the effect of these three variables better, we assembled a new composite variable comprising all the possible mutually exclusive combinations of the original predictors transposed to 12 possible levels where we estimated the prevalence of undertreatment. Estimates ranged from 9.8 (patients already admitted to hospice, receiving adjuvant therapy) to 55.3% (new cases admitted to pain or palliative care centre, not receiving adjuvant treatment). Figure 3 confirms that each of the three variables actually has an effect on the occurrence of undertreatment, and within each type of recruiting centre, the other two variables may stratify patients with different frequency. Nevertheless, patients in a hospice had a lower prevalence (range=9.8–30.4%) than those in oncology centres (16.3–39.3%) and pain and palliative centres (11.8–55.3%).

Table 6 shows the prevalence of patients receiving the treatment that we considered appropriate for each specific condition, and the associations with type of patient and type of centre. Non-compliance with the treatments identified was generally high, with some variability according to the type of patient and the recruiting centre. New cases had higher non-compliance rates in general. In patients with neuropathic pain, non-compliance was higher in oncology centres; in patients with severe worst pain, non-compliance rates were higher in palliative and pain centres.

## Discussion

Advances in diagnosis and therapy have extended the life expectancy of cancer patients, but for most of them, the last part of their life is impaired by pain, depression and other symptoms related to the disease and treatments that become major contributors to suffering. Considerable evidence from clinical experience shows that cancer pain may be controlled in up to 90% cases with available therapies (Ventafridda *et al*, 1987; Grond *et al*, 1996; Mercadante, 1999). The epidemiology of cancer pain and related treatments in Italy is not well documented, but undertreatment is to be expected as strong opioids are prescribed only to a small proportion of eligible patients. (De Conno *et al*, 2005; Osservatorio Nazionale sull’impiego dei farmaci, 2007). This study reporting the results of an observational study produced a significant picture of the management of cancer pain for an unselected population of cancer patients in the care of different Italian specialist facilities (oncology clinics, pain and palliative care and hospice centres). Forthcoming studies will describe the longitudinal change over time of both therapeutic and outcome variables and will compare the effectiveness of different analgesic strategies (Apolone *et al*, 2006b).

The main aim of this analysis was to estimate the quality of analgesic drug regimens across different settings. We applied the PMI as recommended by Cleeland *et al* (1994) using the worst intensity of pain to calculate the score. By changing the pain measure criteria, it yields figures which cannot be fully compared, as suggested by earlier research (de Wit *et al*, 1999).

The prevalence of undertreatment found in this study (25% in the whole sample and up to 55% in some groups) compares the earlier results ranging from as low as 7–9% in a survey in the United Kingdom (Russell *et al*, 2006) to 82% in a sample of non-small cell lung cancer patients entering clinical trials in Italy (Di Maio *et al*, 2004), with the weighted overall mean of 43% across 26 different studies (Deandrea *et al*, 2008).

When the factors associated with negative PMI were examined in this study, undertreatment tended to be lower according to the year of publication, showing a time trend between articles published before and after 2000; it was also associated with socioeconomic and geographic factors: patients coming from Asian countries and patients with lower socioeconomical status had the highest risk of undertreatment. Other factors that predict a negative PMI consistently were having a less advanced disease and the discrepancy between the patient's and physician's assessments of pain intensity (Russell *et al*, 2006; Deandrea *et al*, 2008).

This study shows that the baseline PMI scores are also influenced by the time of referral. Patients just referred to a centre had negative PMI more often (41%) than patients already followed by the centre. This factor has been underestimated in the literature and it should, therefore, be stressed that the patient's pain history is a significant variable in evaluating epidemiological and therapy-related information. A selection bias can also have occurred in our study as physicians may have referred those patients who needed expert advice for treating their pain, although 20% of negative PMI persisted also in patients referred to the centre since a long time (Table 3).

In this study, the setting of care and receiving an adjuvant analgesic drug treatment were also predictors of the PMI status. Being admitted to a hospice and the use of an adjuvant drug were in fact independently associated with more adequate analgesic treatments. These observations might be interpreted as indicative of a delay of starting strong opioids influenced by attitudes of practice and setting of care. Time of patient referral to the centre, considered as very important, is not enough to explain pain and pain treatment history. Organisation and expertise in pain management and monitoring by different specialists and in different settings, such as an in-patient hospice or outpatient pain clinic, probably influence the recourse to more potent drugs.

The use of adjuvants for neuropathic pain is an indicator of compliance with available guidelines (SIGN, 2008), and perhaps its association with more appropriate opioid use is consistent in explaining individual differences among operators even in apparently similar settings.

Although the usefulness of the PMI is proved by the large number of studies that have used this score since 1994, some drawbacks are well known (Deandrea *et al*, 2008) and others may be derived from our analysis. It takes into account only one characteristic of pain (the intensity) and the most potent opioid prescribed, but does not reflect other pain characteristics, opioid titration, route of administration, patient's compliance, rescue and adjuvant therapies, or the use of non-pharmacological therapies. For instance, for patients with neuropathic pain and severe pain, it might be more appropriate to add an adjuvant drug for neurophatic pain rather than changing the type of analgesic drugs or increase its dosage. Eventually, PMI is a static measure that allows cross-sectional evaluation, but cannot adequately assess events over time. Given the longitudinal nature of the study, we were also able to prospectively evaluate the PMI negative status at 4 weeks. As expected, as it is very sensitive to the administration of opioids, the proportion of undertreatment falls substantially approaching 5%, with little variability across variables (type of centre and patient). As a conclusion, PMI can be viewed as a preliminary indicator (a screener) of potential undertreatment that requires a further more specific evaluation.

When we used a selected list of indicators to describe the attitude of Italian physicians towards treating subgroups of patients with specific clinical conditions, we found that the general picture captured by PMI does reflect substantial undertreatment. These indicators also added important information about less than optimal practices. Only 59% of patients with severe worst pain actually received a strong opioid as ‘around the clock therapy’ at the time of study enrolment, 44% with neuropathic pain had an adjuvant drug prescribed and 24% with breakthrough pain had a ‘rescue’ analgesic. The interpretation of the fact that only 38% of patients with bone metastasis actually were receiving bisphosphonates is less straightforward as the role of these drugs to obtain immediate pain relief remains uncertain, despite results from a *Cochrane Review* document that the addition of bisphosphonates can be beneficial (Wong and Wiffen, 2002). Interestingly, also these indicators were associated with the place of care.

In summary, in this prospective observational study, the PMI method indicated a high prevalence of analgesic undertreatment in Italy, around 50% in some subgroups, which varies according to several factors related to the characteristics of the cases and to some structural and organisation variables. These findings confirm the results of a systematic review (Deandrea *et al*, 2008). Other clinical indicators suggest that undertreatment is substantial in some specific subgroups, with underuse of specific adjuvant drugs. Recourse to the WHO third-level drugs is still delayed in a large percentage of patients with cancer pain, half of whom are still treated with anticancer drugs, but very few (31%) had adequate information about prognosis.

It is likely that after the first impact of the WHO guidelines on cancer pain after their first publication in 1986, the present overall strategy for managing cancer pain in Italy would need more structured interventions to improve and standardise quality of care. Evidences about the most effective interventions in changing the outcome of current practice in cancer pain management are scarce. Although guidelines implementation proved to have an impact on patient-reported outcomes (Ventafridda *et al*, 1990; Du Pen *et al*, 1999), educational and quality improvement programmes had no effect in modifying patients’ pain severity, but a beneficial effect was seen as the result of providing specialised palliative care (Goldberg and Morrison, 2007). Others have advocated the efficacy of the implementation of institutional policies, adoption of clinical pathways and pain consultation models (Brink-Huis *et al*, 2008). In Italy where lack of homogeneous service development for patients with cancer pain, cultural barriers and poor guidelines dissemination are likely to exist, a combination of approaches adopted by professional and scientific associations, regulatory authorities and institutions could improve the way in which the pain is managed in this patient population.

Our results also support the idea that palliative care, like the prevention and relief of symptoms in cancer patients, needs to be a component of patient care also during anticancer treatment, and not merely at the end of life. A wider approach is therefore needed, with better education on palliative care and pain management to improve the use of opioids, to standardise the practice of managing cancer pain to minimum standards (Maltoni, 2008; Meyers *et al*, 2004) and to improve the physician–patient communication (Harrington and Smith, 2008).

## Figures and Tables

**Table 1:** Characteristics of patients at baseline (*n*=1801)

**Characteristics**	**%**	**Mean, s.d.**
Age		63.9, 12.1
Female	47.3	
Karnofsky PS, <50	11.5	
		
*Primary tumour*		
Lung	21.8	
Breast	15.9	
Colorectal	13.7	
Prostate	7.9	
Gynecological	6.1	
Pancreas	6.0	
Genitourinary	6.1	
Stomach	5.5	
Head and neck	4.5	
Liver	1.2	
Others	9.9	
Unknown	1.4	
		
Bone metastasis	46.8	
		
Earlier surgery	58.0	
Earlier chemotherapy	65.2	
Earlier hormonotherapy	20.0	
Earlier radiotherapy	40.3	
Others	5.4	
		
Ongoing chemotherapy	49.0	
		
Patients aware of prognosis (reported by physician)	30.3	
		
*Type of recruiting centre*		
Oncology centre	59.4	
Palliative care	17.0	
Pain centre	15.1	
Hospice	7.7	
Others	0.8	
		
*Time of recruiting*		
New cases	25.7	
Already admitted	74.3	
		
*Pain intensity (0–10)*		
Worst (previous week)		6.8, 2.2
Mean (previous week)		4.5, 2.0
Current		3.4, 2.7
Least (previous week)		2.6, 2.0
		
*Pain intensity (according to WHO)*		
None	1.1	
Mild	26.0	
Moderate	65.9	
Severe	7.1	
		
Pain relief (0–100)		55.1, 26.4
		
Patients with breakthrough pain	48.4	
Patients with neuropathic pain	25.7	
		
*Type of analgesic care*		
Around the clock therapy		
None	5.9	
Only NSAID	8.8	
Only weak opioids	10.6	
NSAID with weak opioids	14.1	
One strong opioid	38.3	
NSAID/weak opioid+strong opioid	19.2	
More than one strong opioid	3.1	
		
Rescue therapy		
None	53.2	
Only NSAID	24.9	
Only weak opioids	3.8	
NSAID with weak opioids	2.9	
One strong opioid	11.8	
NSAID/weak opioid+strong opioid	3.2	
More than one strong opioid	0.2	
		
Adjuvant therapy		
Corticosteroids	40.1	
Anticonvulsants	15.8	
Antidepressants	10.8	
Bisphosphonates	18.5	

**Table 2:** Percentages of patients with negative PMI score for different pain items (from Brief Pain Inventory)

		**Time of recruiting**	
**Pain**	**All**	**Already admitted**	**New patients**
Worst pain	25.3	20.4	38.3
Least pain	8.9	6.0	16.7
Current pain	12.2	8.5	22.0
Mean pain	14.6	11.3	23.7
Overall pain (four items)	14.0	11.1	21.8

**Table 3:** Percentages of patients with negative PMI scores according to the type of recruitment (new patients or patients already admitted at the time of inclusion in the study)

	**Days since admission to the centre**			
	**0 (new patients)**	**1–7**	**8–27**	**>27**
*N*	282	171	210	1101
				
*PMI negative scores*				
*n*	126	48	46	222
%	44.7	28.1	21.9	20.2
				
Cochran–Armitage trend test	*χ*^2^=8.124			
	*P*<0.0001			
				
Heterogeneity test	Statistic=73.8092			
	*P*<0.0001			

**Table 4:** Percentages of patients with negative PMI scores according to selected variables

	***N***	**%**	**OR**	**95% CI**	***P*-value**
*Age (years)*					
<51	254	22.8	1.00	—	—
51–60	402	25.4	1.15	0.79–1.66	0.4610
61–70	558	23.5	1.04	0.73–1.47	0.8415
>70	583	28.0	1.31	0.93–1.85	0.1227
					
*Sex*					
Male	947	24.2	1.00	—	—
Female	850	26.5	1.13	0.91–1.40	0.2650
					
*Bone metastasis*					
Yes	842	22.3	1.00	—	—
No	955	27.9	1.34	1.08–1.67	0.0072
					
*Ongoing chemotherapy*					
Yes	883	23.0	1.00	—	—
No	914	27.5	1.27	1.02–1.57	0.0294
					
*Type of recruiting centre*					
Oncology centre	1066	23.7	1.48	0.93–2.35	0.0978
Pain center	271	34.0	2.44	1.47–4.05	0.0006
Palliative care	307	26.1	1.67	1.01–2.78	0.0471
Hospice	138	17.4	1.00	—	—
Others	15	33.3	2.38	0.74–7.58	0.1440
					
*Type of cancer* a					
NSCLC	391	22.5	1.15	0.67–1.98	0.6214
Breast	287	23.7	1.23	0.70–2.15	0.4757
Colorectal	247	31.2	1.79	1.02–3.13	0.0416
Prostate	142	21.1	1.06	0.56–2.00	0.8617
Pancreas	108	24.1	1.25	0.65–2.42	0.5037
Stomach	99	20.2	1.00	—	—
					
*Breakthrough pain*					
Yes	870	24.2	1.00	—	—
No	927	26.4	1.14	0.92–1.41	0.2409
					
*Neuropathic pain*					
Yes	455	24.8	1.00	—	—
No	1314	25.2	1.02	0.80–1.30	0.8806
					
*Analgesic adjuvant therapy*					
Yes	1078	18.1	1.00	—	—
No	719	36.0	2.55	2.05–3.17	<0.0001

a Only cancers with frequency >5%.

**Table 5:** Multivariate association (multivariable logistic regression) between selected variables and PMI negative scores

	**OR**	**95% CI**	***P*-value**
*Age (years)*			
<51	1.00	0.69–1.46	0.9916
51–60	1.04	0.75–1.44	0.8021
61–70	1.00	—	—
>70	1.21	0.91–1.62	0.1870
			
*Sex*			
Male	1.00	—	—
Female	1.21	0.96–1.53	0.1082
			
*Bone metastasis*			
Yes	1.00	—	—
No	1.05	0.82—1.35	0.6795
			
*Ongoing chemotherapy*			
Yes	1.00	—	—
No	1.17	0.91–1.51	0.2195
			
*Type of recruiting centre*			
Palliative care+pain centre	2.09	1.27–3.45	0.0038
Oncology centre	2.09	1.26–3.45	0.0041
Hospice	1.00	—	—
			
*Type of cancer*			
Pancreas	1.00	—	—
Colorectal	1.43	0.82–2.48	0.2047
Gynecologic	1.42	0.80–2.51	0.2282
Others	1.15	0.69–1.91	0.5865
			
*Breakthrough pain*			
Yes	1.00	—	—
No	1.09	0.86–1.37	0.4793
			
*Neuropathic pain*			
Yes	1.14	0.87–1.49	0.3514
No	1.00	—	—
			
*Analgesic adjuvant therapy*			
Yes	1.00	—	—
No	2.40	1.89–3.04	<0.0001
			
*Time of recruiting*			
Already admitted	1.00	—	—
New cases	2.46	1.88–3.20	<0.0001

**Table 6:** Compliance with selected indications according to specific subgroups

		**Type of patient**				**Type of centre**					
	**ALL**	**New patients**	**Already admitted**	**Wald *χ*^2^**	***P*-value**	**Oncology**	**Pain**	**Palliative care**	**Hospice**	**Wald *χ*^2^**	***P*-value^a^**
*Breakthrough pain*											
Rescue (no)											
*n*	663	174	474	5.13	0.0236	367	138	105	53	1.87	0.6008
%	76.2	82.1	73.9			75.5	79.8	76.1	72.6		
											
Rescue (yes)											
*n*	207	38	167			119	35	33	20		
%	23.8	17.9	26.1			24.5	20.2	23.9	27.4		
											
Total	870	212	641			486	173	138	73		
											
*Neuropathic pain*											
Adjuvant (no)											
*n*	252	65	180	3.68	0.0547	154	34	51	13	11.37	0.0099
%	55.4	63.1	52.3			61.8	42.5	53.1	43.3		
											
Adjuvant (yes)											
*n*	203	38	164			95	46	45	17		
%	44.6	36.9	47.7			38.2	57.5	46.9	56.7		
											
Total	455	103	344			249	80	96	30		
											
*Bone metastasis*											
Bisphosphonates (no)											
*n*	522	159	354	34.91	<0.0001	252	108	93	69	50.55	<0.0001
%	62.0	80.3	56.1			52.0	75.5	69.4	86.3		
Bisphosphonates (yes)											
*n*	320	39	277			233	35	41	11		
%	38.0	19.7	43.9			48.0	24.5	30.6	13.8		
											
Total	842	198	631			485	143	134	80		
											
											
*Worst pain* >*7*											
Around the clock therapy (no)											
*n*	327	140	178	25.91	<0.0001	174	64	75	14	12.61	0.0056
%	40.9	53.2	34.2			38.7	43.2	51.0	25.5		
											
Around the clock therapy (yes)											
*n*	473	123	343			276	84	72	41		
%	59.1	46.8	65.8			61.3	56.8	49.0	74.5		
											
Total	800	263	521			450	148	147	55		

a Logistic regression test.

## APPENDIX

**Protocol Writing Committee**

Giovanni Apolone: Istituto di Ricerche Farmacologiche ‘Mario Negri’, Via Giuseppe La Masa 19, 20156 Milan, Italy; Oscar Bertetto: Oncologia Medica, Ospedale Molinette, Corso Bramante 88, 10126 Turin, Italy; Augusto Caraceni: Unità Operativa Cure Palliative, Istituto Nazionale dei Tumori, Via Venezian 1, 20123 Milan, Italy; Oscar Corli: Istituto di Ricerche Farmacologiche ‘Mario Negri’, Via Giuseppe La Masa 19, 20156 Milan, Italy; Franco De Conno: Unità Operativa Cure Palliative, Istituto Nazionale dei Tumori, Via Venezian 1, 20123 Milan, Italy; Roberto Labianca: Oncologia Medica, Ospedali Riuniti di Bergamo, Largo Barozzi 1, 24100 Bergamo, Italy; Marco Maltoni: Hospice Forlimpopoli, ASL Forli Cure Palliative, Via Duca D’Aosta 33, 47034 Forlimpopoli (FC), Italy; Maria Flavia Nicora: Direzione Medica, Grunenthal-Formenti, Via R Koch 1/2, 20152 Milan, Italy; Valter Torri: Istituto di Ricerche Farmacologiche ‘Mario Negri’, Via Giuseppe La Masa 19, 20156 Milan, Italy; Furio Zucco: Hospice Garbagnate, Cure Palliative A.O. Salvini, Viale Forlanini 121, 20024 Garbagnate Milanese (MI), Italy.

**Cancer Pain Outcome Research Study Group Members**

V Adamo: UO di Oncologia Medica e Terapie Integrate, Dipartimento di Patologia Umana, Policlinico G Martino, Messina; M Airoldi: SC Oncologia Medica 2, Ospedale San Giovanni Antica Sede, Torino; M Allegri: Servizio Anestesia, Rianimazione I e Tecniche Antalgiche, Fondazione IRCCS Policlinico San Matteo, Pavia; G Altavilla: Oncologia Medica, Dipartimento di Patologia Umana, Università degli Studi di Messina; G Azzarello: UOC Oncologia ed Ematologia Oncologica, ASL 13 Regione Veneto, Noale (VE); E Bandieri: Operative Unit Medical Oncology, Carpi Mirandola Hospital, Modena; ML Barzelloni: UO Oncologia, PO ‘G da Procida’, ASL SA 2, Salerno; F Bassan: UO di Oncologia, Az ULSS n. 4 Regione Veneto, Ospedale di Thiene; G Belfiore: Hospice–Cure Palliative, ASL Chieti; G Bernardo: Oncologia Medica II, Fondazione Salvatore Maugeri (FSM–IRCCS), Pavia; G Bersano: SOC Oncologia, ASL9, Ivrea (TO); R Bertè: UO Oncologia Medica, AO ‘Guglielmo da Saliceto’, Piacenza; O Bertetto: Centro Oncologico Ematologico Subalpino (COES), Ospedale Molinette, Torino; C Bianchessi: UO Medicina Generale, Ospedale Maggiore di Crema; A Bologna: Servizio di Oncologia, Arcispedale Santa Maria Nuova, Reggio Emilia; S Bravi: UO di Oncologia, ASL n. 1 dell’Umbria, Città di Castello; A Buono: Unità Cure Palliative, AUSL 12 Viareggio (LU); F Buzzi: Oncologia Degenza, Azienda Ospedaliera Terni; C Cacioppo: Hospice SM delle Grazie, Fondazione Don Gnocchi, Monza; J Capuccini: UO Terapia del Dolore e Cure Palliative, ASUR Marche-ZT3, Fano (PU); C Caroti: Osteoncologia e Cure Palliative, SC Oncologia Medica, EO Ospedali Galliera, Genova; G Castagnini: Unità Cure Palliative e Terapia del Dolore, AO San Gerardo, Monza; M Ceste: SOC Oncologia, Ospedale Cardinal Massaia, Asti; S Chisari: Centro di Medicina del Dolore, AO Universitaria ‘Vittorio Emanuele, Ferrarotto e S Bambino’, Catania; B Chiurazzi: Divisionene di Oncologia, AORN ‘A Cardarelli’, Napoli; A Comandone: Oncologia, AO Gradenigo, Torino; E Cortesi: Oncologia Medica B, Policlinico Umberto I, Università ‘Sapienza’, Roma; C Crispino: UOS di Terapie di Supporto, AO ‘Monaldi’, Napoli; A Cuomo: UOD Terapia Antalgica, Istituto Nazionale Tumori ‘Fondazione Pascale’, Napoli; A De Martino: UO di Medicina del Dolore e Cure Palliative, Hospice ‘Giardino dei Girasoli’, DS B di Eboli, ASL Salerno 2; A De Matteis: Oncologia Medica C, Istituto Nazionale Tumori ‘Fondazione Pascale’, Napoli; A De Salve: Unità di Cure Palliative e Terapia del Dolore, Ospedale Bassini, AO San Gerardo di Monza; S Del Prete: UOC Oncologia, Ospedale ‘San Giovanni di Dio’, Frattamaggiore (NA); F Della Rovere: UOS Terapia Antalgica e Cure Palliative, AO Universitaria San Martino, Genova; C Dello Ioio: Assistenza Domiciliare Oncologica Integrata, ASL Salerno 1, Salerno; S Derni: Unità Cure Palliative, Dipartimento Oncologico, AUSL Forlì; L Di Alesio: Dipartimento Oncologico, POS Andrea Felettino, ASL n. 5 Regione Liguria, La Spezia; F Di Costanzo: Struttura Oncologica Complessa, A.O. Universitaria Careggi, Firenze; C Di Fonzo: UOC Oncologia Medica Ospedaliera, Ospedale ‘Santa Maria Goretti’, Latina; R Di Gregorio: Terapia Antalgica, Ospedale Sacro Cuore di Gesù Fatebenefratelli, Benevento; M Di Lanno: Servizio Oncologia, Ospedale ‘San Giuliano’, Distretto 58 ASL NA 2, Giugliano in Campania (NA); M Di Seri: UOC Oncologia A, Policlinico Umberto I, Università ‘Sapienza’, Roma; M Di Stefano: Anestesia e Rianimazione, Centro Tumori Ascoli; D Dini: Terapie Palliative – Hospice, Centro Oncologico AO Modena; D Dini: SC Terapia Antalgica e Riabilitazione, Istituto Nazionale per la Ricerca sul Cancro, Genova; M Duro: UO di Oncologia, Ospedale Valduce, Como; G Facchini: Oncologia Medica B, Istituto Nazionale Tumori ‘Fondazione Pascale’, Napoli; A Farris: Oncologia Medica Universitaria, Sassari; C Ferrara: UO di Oncologia Medica, AO ‘San Giuseppe Moscati’, Avellino; P Ferrari: UO Cure Palliative, Fondazione Maugeri, Ospedale San Martino, AO Provincia di Pavia, Mede (PV); M Frascaroli: Riabilitazione Oncologica, Fondazione Maugeri IRCCS, Pavia; D Furiosi: USS Terapia Antalgica e Cure Palliative, AO della Provincia di Lodi; F Fusco: UOS Cure Palliative Polo Ponente, ASL n. 3 Genovese, Genova; V Gebbia: UO Oncologia Medica, Dipartimento Oncologico III livello, Casa di Cura di Alta Specialità ‘La Maddalena’, Palermo; MG Ghi: Oncologia Medica, Azienda ULSS n. 12, Venezia Mestre; D Giannunzio: UO Cure Palliative, Azienda Istituti Ospedalieri, Cremona; C Giuffrè: Divisione di Oncologia Medica, AO Bianchi Melacrino Morelli, Reggio Calabria; C Guarino: II UOC di Pneumologia Oncologica, AO ‘Monadi’, Napoli; F Henriquet: Associazione Gigi Ghiotti ‘ONLUS’ – Assistenza domiciliare e in Hospice, Genova; L Isa: Divisione Oncologia Medica, Ospedale ‘Serbelloni’, Gorgonzola (MI); P La Ciura: SC Cure Palliative e Hospice, ASL CN 1, Cuneo; F La Rocca: Unità Funzionale Cure Palliative, ASL 6 Livorno; R Labianca: UO di Oncologia Medica, Ospedali Riuniti di Bergamo; C Longhi: UOS Cure Palliative Domiciliari – Hospice ‘Il mantello, Struttura di Mariano Comense, AO Sant’Anna Como; G Lupo: Oncologia Medica e Terapie Innovative, Dipartimento di Patologia Umana, Università degli Studi di Messina; S Mameli: UO Medicina del Dolore, Ospedale ‘A Businco’, Cagliari; A Manni: Hospice Casa Madonna dell’Uliveto, Albinea (RE); L Manzione: UO Oncologia Medica, AO San Carlo, Potenza; C Martini: Hospice, Istituto Nazionale Tumori, Milano; A Martoni: Oncologia Medica, AO Universitaria, Policlinico S Orsola-Malpighi, Bologna; N Marzano: UO Oncologia, Ospedale ‘San. Paolo’, ASL/BA, Bari; S Masseroni: Oncologia, AO ‘S Carlo Borromeo, Milano; V Mattioli: UO di Medicina del Dolore e Cure Palliative, IRCCS Istituto Tumori ‘Giovanni Paolo II’, Bari; P Maurizi: Unità di Cure Palliative–Sez. Dipartimentale del Dip.to Oncologico, Azienda ULS 8, Arezzo; D Miotti: UO di Cure Palliative e Terapia del Dolore, Fondazione Salvatore Maugeri–IRCCS, Pavia; L Montanari: UO Oncologia Medica, Ospedale di Lugo, AUSL Ravenna; L Moscetti: UOC di Oncologia, Ospedale Belcolle, ASL Viterbo; V Murgia: Oncologia Medica, Ospedale Santa Chiara, Trento; LF Nardi: UO Terapia del Dolore e Cure Palliative, Zona territoriale 9, ASUR Marche, Macerata; F Narducci: Oncologia Medica, Ospedale SS Trinità, Sora (FR); F Negri: Divisione di Medicina e Oncologia Medica, Azienda Istituti Ospedalieri, Cremona; C Orlandini: UO Oncologia Medica, AO Universitaria Pisana, Pisa; D Ottaviani: Centro Universitario di Ricerca Oncologica, Ospedale Molinette, Torino; G Pancera: Medical Oncology Department, Igea Hospital, Milan; F Paoletti: Servizio Aziendale Cure Palliative–Hospice, USL n. 2 dell’Umbria, Assisi; A Pappalardo: UO di Oncologia Medica, Centro Clinico e Diagnostico ‘GB Morgagni’, Catania; MK Paris: Oncologia Medica, Polo Oncologico di Biella; C Pittureri: Servizio Cure Palliative–Hospice, AUSL Cesena, Savignano sul Rubicone (FC); L Piva: Unità di Cure Palliative, UO Oncologia Medica, AO San Paolo, Milano; L Ponchio: Divisione di Oncologia Medica I, Fondazione Salvatore Maugeri, Pavia; C Reale: UOC Anestesia e Rianimazione C, Policlinico ‘Umberto I’, Università ‘Sapienza’, Roma; A Rinaldi: UO Oncologia, OC Castellaneta, AUSL Taranto Polo Occidentale; E Romagnoli: UO Oncologia, Ospedale Civile di Macerata; A Russo: Oncologia Medica, Policlinico Universitario ‘P Giaccone’, Palermo; C Sandomenico: Oncologia Medica A, Istituto Nazionale Tumori ‘Fondazione Pascale’, Napoli; A Sbanotto: Unità di Terapie di Supporto e Cure Palliative, Istituto Europeo di Oncologia, Milano; F Scanzi: UO Oncologia, IRCCS Multimedica, Sesto S Giovanni (MI); I Schiavetto: Oncologia Medica Falk, Ospedale Niguarda ‘Ca’ Granda’, Milano; L Trentin: UO Terapia del Dolore e Cure Palliative, Az ULSS n. 6 Ospedale San Bortolo, Vicenza; M Tucci: AO Universitaria San Luigi, Orbassano, Torino; G Vaccaro: Anestesia e Rianimazione, UOS di Terapia del Dolore, PO ‘Abele Ajello’, Mazara del Vallo (TP); D Valenti: Fondazione Hospice MTC Seragnoli, Bentivoglio, Bologna; VM Valori: Divisione di Oncologia, Casa Sollievo della Sofferenza, San Giovanni Rotondo (FG); G Vasini: UO Oncologia Medica, AO Universitaria, Parma; E Veltri: Centro Oncologico ‘Ettore MS Conti’, Presidi Ospedalieri di Gaeta e Terracina, ASL Latina; V Zagonel: UOC Oncologia, Ospedale ‘San Giovanni Calibita’, Isola Tiberina, Roma; E Zecca: Unità di Cure Palliative (Terapia del Dolore – Riabilitazione), Istituto Nazionale Tumori, Milano; G Zeppetella: UOC di Fisiopatologia, terapia del dolore e cure palliative, AO San Sebastiano, Caserta; M Ziliotti: Cure Palliative, Presidio Ospedaliero di Fidenza; F Zucco: Unità Cure Palliative e Terapia del Dolore, AO ‘G Salvini’, Garbagnate Milanese (MI).
